# *Correspondence:* Some general points regarding Ledberg and Wennberg, BMC Medical Research Methodology 2014 April 27;14:58

**DOI:** 10.1186/s12874-015-0043-9

**Published:** 2015-07-07

**Authors:** Dankmar Böhning, Peter G.M. van der Heijden

**Affiliations:** Southampton Statistical Sciences Research Institute, Mathematics and Medical Statistics, University of Southampton, Southampton, SO17 1BJ UK; Southampton Statistical Sciences Research Institute, University of Southampton, Southampton, SO17 1BJ UK; Department of Methods and Statistics, Social Sciences, University of Utrecht, Utrecht, Netherlands

## Abstract

The purpose of this note is to contribute some general points on a recent paper by Ledberg and Wennberg (BMC Med Res Meth 14:58, 2014) which need to be rectified. They advocate the capture-removal estimator. First, we will discuss drawbacks of this estimator in comparison to the Lincoln-Petersen estimator. Second, we show that their evaluation of the Chao estimator is flawed. We conclude that some statements in Ledberg and Wennberg with respect to Chao’s estimator and removal estimation need to be taken with great caution.

## Main text

In a recent paper, Ledberg and Wennberg [1] propose to use the capture-removal estimator (Otis et al. [2]; Seber [3]; Borchers et al. [4]; ch. 5) for estimating the size of a hidden population from register data. It is assumed that a register has registrations from *M* occasions with *M*>1. These occasions refer to different points in time so that they are chronologically ordered. The approach, at any occasion, consists of considering only new registrations and ignore those that have been identified before. Under the assumption that registration is *independent* at occasions and probability of registration is *homogeneous* a likelihood function can be determined and maximized in the two parameters involved, the probability of registration and the size *N* of the population. Below we will first show, for two occasions, that the capture-removal estimator can have drawbacks in comparison with the Lincoln-Petersen estimator. Then we will show that the evaluation of the Chao estimator, given by Ledberg and Wennberg, is flawed.

### *M*=2 occasions

We consider the case of two occasions, *M*=2. This is the simplest possible case and also allows an easy comparison with the Lincoln-Petersen estimator and the bias-corrected Chapman estimator (Borchers et al. [4]). Let, as in Ledberg and Wennberg [1], denote with *n*_1_ all registrations at occasion 1 (here every occasion is a new registration) and with *n*_2_ all registrations at occasion 2 that were not yet registered at occasion 1. For the setting of *M*=2 occasions it is possible to derive the maximum likelihood estimate of *N* in a closed form expression: $ \hat N_{R} = \frac {{n_{1}^{2}}}{n_{1}-n_{2}}$, assuming that *n*_1_>*n*_2_ which may or may not be met in practice. We denote this estimator as $\hat N_{R}$, index *R* for removal. For comparison, we consider the Lincoln-Petersen estimator given as $ \hat N_{\textit {LP}} = \frac {n_{1}(m+n_{2})}{m}$ and the Chapman estimator given as $ \hat N_{\textit {Ch}} = \frac {(n_{1}+1)(m+n_{2}+1)}{(m+1)}$.

We will show that the latter two estimators are the better choice in the following two situations: first, when the assumption of constant and occasion-independent inclusion probabilities of the capture-removal estimator are met, the Lincoln-Petersen and the Chapman estimators are generally more efficient. Second, when the assumption of homogeneous inclusion probabilities that underlies the capture-removal estimator is not met, the capture-removal estimator is biased whereas the Lincoln-Peterson and the Chapman estimators are not. However, when there is behavioral response, i.e. after an inclusion the probability of the next inclusion increases, the Lincoln-Peterson and the Chapman estimators are biased downwards whereas the capture-removal estimator might be less biased depending on the constellation of marginal distributions and occasion dependency. We note that in the biological literature the first condition is known as *M*_0_ for the inclusion probability being constant over time (under which the removal estimator is derived) and the second condition is known as *M*_*t*_ for the inclusion probability varying with occasions (under which the Lincoln-Petersen and Chapman estimators are derived), whereas behavioral response is *M*_*b*_.

To illustrate we have done the following simulation study. The population size is *N*=1000 and we looked at different scenarios of registration probability. Let *p*_1_ be the registration probability at occasion 1 and *p*_2_ at occasion 2, registration is independent from occasion. Table 1 shows the mean and standard deviation of the Lincoln-Petersen, the Chapman and the removal estimator, respectively, for different settings. All simulation results are based upon 1000 replications. In setting 1 and 2 of Table 1 we look at equal registration probabilities, but the second setting has smaller ones. Both give reasonable mean results but the Lincoln-Petersen and Chapman estimators have the smaller standard deviation. Also, the variance of both estimators increase if the registration probability decreases. For setting 3 and 4 of Table 1, registration probability for occasion 2 is larger than for occasion 1. Here, the capture-removal estimator overestimates, in particular for setting 4 where it becomes almost useless. On the contrary, for setting 5 and 6 of Table 1, where the registration probability for occasion 2 is lower than for occasion 1, the capture-removal estimator underestimates, for setting 6 quite seriously. In all six settings, the Lincoln-Petersen and the Chapman estimators are giving unbiased estimates although the variance increases when the registration probabilities become small. For fairness, we also show simulation results for two settings where the removal estimator is doing better than the Lincoln-Petersen and Chapman estimators. This is in the case of behavioral response, i.e. once observed for the first time the probability for a second observation increases. In setting 7 at the first occasion is 0.50, but at the second occasion it increases to 0.30/(0.30+0.20) = 0.60 for those who have been already observed. In setting 8 this second conditional probability increases from 0.50 to.375/(.375+.125) = 0.75. In these two situations the removal estimator outperforms the Lincoln-Petersen and Chapman estimators. The last two settings 9 and 10 are two further examples of behavioral response and show that also the capture-removal estimator can be seriously biased. The reason is that the capture-removal estimator, at least in the way it is used by Ledberg and Wennberg, requires that the conditional probability for capture at occasion 2 given no capture at occasion 1 has to be identical to the unconditional probability of capture at occasion 1 (see Borchers et al. [4], p.76) which is not fulfilled in these last two settings.

**Table 1:** Simulation results for registration system with two occasions. *p*_11_ is the probability for capture at occasion 1 and occasion 2, *p*_10_ is the probability for capture at occasion 1 but not at occasion 2, and so forth. The marginal probabilities for capture at occasion 1 and 2 are *p*_1_=*p*_11_+*p*_10_ and *p*_2_=*p*_11_+*p*_01_, respectively. In settings 1 to 6 inclusion on occasion 1 is independent of inclusion on occasion 2. In settings 7 and 8, occasions become dependent (odds ratio larger than 1) but the conditional probability for capture at occasion 2 given no capture at occasion 1 is identical to the unconditional probability for capture at occasion 1, the capture-removal estimator works fine. In settings 9 and 10, those conditional and unconditional probabilities are different and the capture-removal estimator breaks down

							LP		Chapman		Removal	
Setting	*p*_1_	*p*_2_	*p*_11_	*p*_10_	*p*_01_	*p*_00_	$\bar N_{\textit {LP}}$	SD	$\bar N_{\textit {Ch}}$	SD	$\bar N_{R}$	SD
1	0.5	0.5	0.25	0.25	0.25	0.25	1001	31	1000	31	1007	59
2	0.3	0.3	0.09	0.21	0.21	0.49	1006	77	1000	76	1064	262
3	0.5	0.6	0.30	0.20	0.30	0.20	1002	27	1001	27	1271	113
4	0.3	0.35	0.105	0.195	0.245	0.455	1004	66	1000	65	1825	6475
5	0.5	0.3	0.15	0.35	0.15	0.35	1000	48	998	47	714	21
6	0.3	0.1	0.03	0.27	0.07	0.63	1021	155	999	146	392	17
7	0.5	0.55	0.30	0.20	0.25	0.25	955	28	955	28	1003	57
8	0.5	0.625	0.375	0.125	0.25	0.25	834	18	834	18	1009	56
9	0.3	0.1	0.065	0.235	0.035	0.665	464	34	462	34	340	15
10	0.5	0.5	0.4	0.1	0.1	0.4	625	17	626	17	625	17

### Multiple occasions

We would like to make a second point considering *M* occasions. In the case of homogeneity and independence the probability of observing exactly *y* registrations for a unit is given by the binomial $P(Y=y) = {M \choose y} p^{y} (1-p)^{M-y}$, where *p* is the probability of a registration at any, fixed occasion. Then, the Chao estimator (Chao [5]) of hidden units, the frequency of units with exactly zero registrations, is given as $ \hat f_{0} = \frac {M-1}{M}\frac {{f_{1}^{2}}}{2f_{2}}$, which is asymptotically unbiased where asymptotics refer to *N* and *f*_*x*_ is frequency of units with exactly *x* registrations. If *M* is large it becomes the Chao estimator under Poisson sampling that is mentioned in Ledberg and Wennberg [1]. Clearly, the asymptotic unbiasedness result holds under homogeneity for any *M*, although the variance is smaller the larger *M* is. There is also a biased-corrected version of Chao’s estimator which reduces the small-sample bias under homogeneity, but for the sake of brevity we will not consider this bias-corrected estimator here. If there is heterogeneity in registration (this is referred to in the biological literature as *M*_*h*_) then the result in Chao ([5, 6]) says that the estimator represents only *a lower bound*. This is also why Chao’s estimator is also called the *lower bound* estimator. The underestimation bias is mostly small in comparison with other estimators that assume homogeneity. When *M* is becoming larger the underestimation bias will also become smaller in an absolute sense. This occurs since the number of registered users $s= \sum _{i=1}^{M} f_{i}$ will become larger and the number of units not observed becomes smaller. This is because the probability of a unit remaining undetected will decrease with *M* becoming large as can be seen from the following equation (binomial sampling under heterogeneity) $P(Y=0) = \sum _{j=1}^{J} (1-p_{j})^{M} w_{j} \rightarrow 0$, as *M* approaches *∞*, where *p*_*j*_ is the probability of registration in subpopulation *j* and *w*_*j*_ its associated subpopulation weight. Hence the following statement in the discussion of Ledberg and Wennberg [1] is unsound and needs to be revised:

Assume that registrations are followed over a period of time. Since estimates obtained by Chao’s estimator should not strongly depend on the duration of the time period used, similar estimates should be obtained if the first half of the time period is used compared to if the whole time period is used.

Clearly, in the case of heterogeneity, the bias of Chao’s estimator is smaller when a larger number of occasions is considered. To illustrate this point we have done a small simulation experiment. The true *N* is 200 and all results are based upon 1000 replications. In the first setting, the population is homogeneous with *p*=0.1. In the second, setting we assume a subpopulation structure with equal weights *w*_*j*_=0.5 allocated to *p*_1_=0.3 and *p*_2_=0.05. In Table 2, $\bar s$ denotes the mean of the number of observed different users (averaged over the 1000 replications), $\bar f_{0}$ denotes the mean of the estimated frequency of hidden units *f*_0_ (averaged over the 1000 replications), and SD($\hat f_{0}$) its estimated standard deviation. For an unbiased estimate we should have that $\bar s+ \bar f_{0}= N$, in our case 200. For setting 1, this is practically the case, although the standard deviation is better for *M*=10 in comparison to *M*=5. For setting 2, the estimator experiences bias, with a value of 54 for *M*=5 and a value of 34 for *M*=10, the latter being clearly smaller than the former. The reason for this bias is that the lower bound of Chao’s estimator will only be reached under homogeneity and in setting 2 there is heterogeneity. In such a practical situation it pays off to have a longer observation period. However, if the observation period is taken to be too long, the violation of the assumption of a closed population may become more likely.

**Table 2:** Chao’s estimator for registration system with *M* occasions and true *N*=200

			*M*=5				*M*=10			
Setting	*p*_1_	*p*_2_	$\bar s$	$\bar f_{0}$	$\bar N$	SD($\hat f_{0}$)	$\bar s$	$\bar f_{0}$	$\bar N$	SD($\hat f_{0}$)
1	0.1	0.1	82	120	202	52	130	70	200	20
2	0.3	0.05	106	40	156	13	137	29	166	10

We conclude that the capture-removal estimation approach can be useful under certain (but not all) constellations of behavioral response. However, it is sensitive to violations of registration homogeneity and independence of occasions, as pointed out in Borchers *et al.* ([4]; Ch. 5). If the Lincoln-Petersen approach can be used instead it is the better choice for two reasons: it does not require identical registration probabilities in the occasions (the marginal distributions in Table 3 do not need to be equal), and secondly, makes full use of the available information in Table 3 (the removal estimator uses only the marginal information for occasion 1), so that the Lincoln-Petersen estimator has the better efficiency.

**Table 3:** Registration system with two occasions

			Occasion 2		
		1		0	
	1	*m*		*n*_1_−*m*	*n*_1_
Occasion 1					
	0	*n*_2_		*x*	
		*m*+*n*_2_			*N*

It has been seen that some statements in Ledberg and Wennberg on Chao’s estimator, in particular on its independence of the number of occasions, need to be revised, especially, if there is population heterogeneity. Even if there is homogeneity the variation for the entire period will be considerably smaller than for the first half-period. It might be better to compare the Chao estimator for different periods of equal size.

From our perspective, Chao’s estimator remains as one of the most useful estimators in the area. We recently proposed a generalization of Chao’s estimator that can take covariates into account (Böhning *et al.* [7]). Thus observed population heterogeneity can be modelled and the lower bound provided by this covariate adjusted estimator will be closer to the true population size than the unadjusted estimator.
