# Erratum to: *Correspondence*: Some general points regarding Ledberg and Wennberg, BMC Medical Research Methodology 2014 April 27;14:58

**DOI:** 10.1186/s12874-015-0065-3

**Published:** 2015-09-25

**Authors:** Dankmar Böhning, Peter G M van der Heijden

**Affiliations:** Southampton Statistical Sciences Research Institute, Mathematics and Medical Statistics, University of Southampton, Southampton, SO17 1BJ UK; Southampton Statistical Sciences Research Institute, University of Southampton, Southampton, SO17 1BJ UK; Department of Methods and Statistics, Social Sciences, University of Utrecht, Utrecht, Netherlands

## Erratum

The original version of this article [1] unfortunately contained a mistake. The author’s response was missing in both the HTML version of this article. The author’s response is given below.

**Response**

Anders Ledberg* and Peter Wennberg

*Correspondence: anders.ledberg@sorad.su.se

Centre for Social Research on Alcohol and Drugs, SoRAD Stockholm University, SE-10691 Stockholm, Sweden

Full list of author information is available at the end of the article

**Introduction**

We are happy about the attention our publication “Estimating the size of hidden populations from register data” [1] has received and would like to use this opportunity to clarify what our paper is about and what it is not about.

**What our paper is about**

In our paper we are considering the problem of estimating the size of an incompletely sampled population. The particular case we have in mind is that when a given individual in the population has constant probability, per unit time, of being first registered, but once registered the probability of future registrations might change, perhaps radically. (We use ‘registered’ in a general sense here; the analogous concept in the ecological literature would be ‘captured’, or ‘trapped’). This case is of interest to us since we believe that it could serve as an approximate model for epidemiological data. As an example, consider the “population” of heavy drug users. Assume that there is a constant probability that heavy drug use leads to contact with the health care system for the first time (and a registration). One possible outcome of such a contact is that the client enters a treatment program that implies regular contacts with the health care system (for example methadone maintenance treatment). Consequently, the probability that this particular individual is registered again is very high (close to one). Indeed, that the probability of registration is history dependent seems to us a generic feature of this type of data. In the literature on population estimation in ecology this history dependence is often called behavioral response [e.g. 2]. In keeping with this terminology (of [2]) we call this scenario Model M_b_. In other words, our paper suggests modeling (some types of) epidemiological data using Model M_b_, and to use the maximum likelihood estimator derived under this model [3].

In our paper we evaluate the performance of this maximum likelihood estimator under the scenario we consider, and show when it is applicable, and when it is not (Figure 2 in [1]). In particular, we show that for the estimator to be useful a certain fraction of the population should be sampled, and this fraction depends on the total size of the population (Figure 2 in [1]). An important result is that the estimator is robust under moderate heterogeneity with respect to the probabilities of first registration of different individuals, i.e. they need not be identical for the estimator to be useful (see Figure 3 in [1]). Another contribution is that we show that some other estimators, that have been used on data that could be reasonably modeled using Model M_b_, can have a substantial bias when applied to data from Model M_b_. In particular, we show that an estimator that can be derived assuming that the data follow a truncated Poisson distribution, can have a substantial bias, and that this bias can be positive, i.e. it might lead to an overestimation of the population size (see Figure 6 in [1]).

**What our paper is not about**

Estimating the size of hidden populations is a problem that has been treated by many authors and there are many different methods in use. The basic idea in deriving a measure (an estimator) is to start with a particular scenario (model) for the registrations, and from this model derive an estimator. Thus, key aspects of a real situation (e.g. drug users interacting with the health care system) are captured in an idealized model (Model M_b_ in our case), and given this model an estimator is derived (maximum likelihood estimator in our case). The estimator is then strictly valid only under the model considered. We certainly do not suggest that the maximum likelihood estimator should be used if the data at hand are better described by other models (such as Models M0 or Mt, for example). Indeed, that an estimator derived under model A does not perform well when applied to data generated under model B is neither surprising nor informative for its performance under model A.

Our paper does not provide an evaluation of other estimators, and our evaluation of the maximum likelihood estimator is done only under some particular scenarios. We have no particular attachment to the estimator we propose but for the type of data we are interested in it still seem a most reasonable choice (given, of course, that a suffcient fraction of the population is sampled). Böhning and van der Heijden do not suggest another estimator that works better in this case, something we interpret as them being in tacit agreement with us. Perhaps contrary to these workers, we do not believe in a “universal estimator” that should always be used. Rather, as we suggest in our paper, application of several estimators, relying on different assumptions, might provide complementary information about the data at hand and might help in getting more reliable estimates.

**Competing interests**

The authors declare that they have no competing interests.

**Acknowledgements**

This research has been financed by the Swedish Council for Working Life and Social Research (FAS 2006–1523).

**References**

1. Ledberg, A., Wennberg, P.: Estimating the size of hidden populations from register data. BMC Med Res Methodol 14(58), 58 (2014)

2. Otis, D., Burnham, K., White, G., Anderson, D.: Statistical-Inference From Capture Data On Closed Animal Populations. Wildlife Monogr (62), 7–135 (1978)

3. Moran, P.: A Mathematical Theory Of Animal Trapping. Biometrika 38(3–4), 307–311 (1951)

The original article has been updated to include this.
